# Use of anthropogenic linear features by two medium-sized carnivores in reserved and agricultural landscapes

**DOI:** 10.1038/s41598-017-11454-z

**Published:** 2017-09-14

**Authors:** Georgina E. Andersen, Christopher N. Johnson, Leon A. Barmuta, Menna E. Jones

**Affiliations:** 1 School of Biological Sciences, University of Tasmania, Private Bag 55, Hobart, TAS 7001 Australia; 2 Australian Research Council Centre of Excellence for Australian Biodiversity and Heritage, University of Tasmania, Hobart, TAS 7001 Australia

## Abstract

Many carnivores are threatened by habitat loss and fragmentation. These changes create linear features and habitat edges that can facilitate foraging and/or travel. To understand the significance of anthropogenic linear features in the ecology of carnivores, fine-scaled studies are needed. We studied two medium-sized carnivores: the endangered Tasmanian devil (*Sarcophilus harrisii*) and the near threatened spotted-tailed quoll (*Dasyurus maculatus*), in a mixed landscape of conservation and agricultural land. Using GPS tracking, we investigated their use of intact habitat *versus* linear features such as roads, fences and the pasture/cover interface. Both species showed a positive selection for anthropogenic linear features, using the pasture/cover interface for foraging and roads for movement and foraging. Devils travelled along fence lines, while quolls showed little preference for them. Otherwise, both species foraged in forest and travelled through pasture. While devils and quolls can utilise anthropogenic linear features, we suggest that their continued survival in these habitats may depend on the intensity of other threats, e.g. persecution, and providing that sufficient intact habitat remains to sustain their ecological needs. We suggest that the management of both species and probably many other species of carnivores should focus on controlling mortality factors associated with human use of landscapes.

## Introduction

Carnivores are vulnerable to habitat loss and fragmentation because they typically have low population densities, large area requirements, and are often persecuted by humans^1, 2^. Anthropogenic landscape alteration creates linear features which can have complex effects on carnivores, such as the ecotone between native vegetation and livestock pasture, roads, fences, and power lines. Negative effects can derive from roads and fences which obstruct movement^3–5^. Roads can also increase mortality from vehicle collisions, increase hunting and poaching by providing access to previously inaccessible habitats, and can cause stress due to noise and visual stimuli^4, 5^.

Linear features can also benefit carnivores. Roads and tracks may facilitate travel, enabling foraging carnivores to move further, faster^6, 7^. Roads, fences and power lines can also provide opportunities for hunting and scavenging, because they create edges and barriers that trap prey and furnish carcasses of animals that have died in collisions with vehicles or power lines^8, 9^. Agricultural landscapes frequently contain remnant patches of native vegetation that create edges; these are often rich in small vertebrates and thus concentrate prey for medium-sized carnivores^10, 11^. Many carnivore species, which have become invasive outside their native ranges, flourish in human-altered habitats. This is true of red foxes (*Vulpes vulpes*) and feral cats (*Felis catus*), and is also the case for some carnivores in their native range, including red foxes, raccoons (*Procyon lotor*) and grey foxes (*Urocyon cinereoargenteus*)^12, 13^.

Understanding how anthropogenic landscapes, and particularly the linear features they often contain, affect movement, foraging efficiency and ultimately survival is especially important for threatened and declining species. Around a quarter of extant mammalian species are threatened with extinction and the extinction risk has accelerated over the last forty years^14^. To evaluate the significance of linear features in the ecology of carnivores, we need detailed behavioural studies showing the extent to which they exploit or avoid them.

We examined the habitat use and fine-scaled movement within home ranges (i.e. third-order resource selection)^15^ of two medium-sized carnivores in a mixed conservation and agricultural landscape in Tasmania, Australia. The Tasmanian devil (*Sarcophilus harrisii*) is a hypercarnivorous pounce-pursuit predator and specialist scavenger with morphological specialisations for eating bone^16^. It weighs 5–14 kg and has a large home range (2145 ha; G. Andersen, unpublished data). The spotted-tailed quoll (*Dasyurus maculatus*) is a hypercarnivorous pounce or ambush predator^16^, that has specialised morphological adaptions to utilise arboreal habitats. It weighs 0.9–5 kg and has a smaller home range than devils (528 ha; G. Andersen, unpublished data). Both are diet generalists but primarily feed on mammals^17^. At our study site in northwest Tasmania, the Tasmanian devil is not yet affected by Devil Facial Tumour Disease (DFTD), that has caused severe population decline elsewhere in its range^18^ leading to its Endangered status on the IUCN red list^19^. In Tasmania, the north western part of the state is the stronghold for the spotted-tailed quoll, which is listed as Near Threatened on the IUCN red list^20^. To investigate the habitat use of sympatric devils and quolls, we examined only nocturnal habitat use, as while quolls can be active during the day^21^, devils are predominately nocturnal^22^. We asked the following questions: (1) how do these species utilise landscapes modified by agricultural land use? (2) to what extent do they use edges between pasture and natural vegetation? And, (3) how do roads and tracks affect movement?

## Results

We GPS-collared seven adult devils (three males and four females) and four adult quolls (two males and two females) between November 2012 and February 2013, and twelve adult devils (six males and six females) and six adult quolls (five males and one female) between October 2013 and January 2014. All animals were re-trapped and their collars removed before the degradable link had corroded. One devil was collared in both years and its data in the second year were not included in analyses. Devils were collared on average for 50 days (range: 40–77 days) and quolls for 28 days (range: 10–49 days). A total of 26 361 successful GPS fixes were obtained, 19 549 for devils and 6812 for quolls. The overall GPS success rate (i.e. the number of successful fixes by an individual GPS collar in proportion to the total number of programmed fixes) was 60 ± 13% (mean ± SD) following the removal of zero fixes and GPS errors. The GPS success rate was higher for quolls (69.9 ± 11%) than devils (49.51 ± 7%). The GPS success rate for the stationary collar in scrub/heath and grass was 95%, 92.5% in pasture, and 87.5% in forest. Higher temperatures that indicated the animal was in a den were recorded on the collars for 49.95% of the devil zero fixes (temperatures over 31.3 °C) and for 23.04% of the quoll zero fixes (temperatures over 33.5 °C) (Supplementary Fig. S1). The GPS collars accurately recorded ambient temperature 74% of the time.

### Habitat Selection

The final model set of habitat selection for devils included two models; the model with all covariates except for ‘Veg same’ (ω_i_ = 73%) and the full model (ΔAIC = 1.92; ω_i_ = 27%). Model-averaged parameter estimates revealed that devils did not select natural vegetation over pasture except for male devils which selected forest over pasture (Table 1 and Supplementary Table S1). They were eight and a half times more likely to be along a sealed road, six times more likely to be along an unsealed road and almost three times more likely to be along a 4WD track than away from a road (Table 1). Devils were ten times more likely to be along a fence and more than two and a two third times more likely to be along a pasture/cover ecotone than away from an ecotone (Table 1). Male and female devils showed a similar positive selection for all road and ecotone types (Supplementary Table S1). In addition, devils regardless of sex selected to be close to their core area (Table 1 and Supplementary Table S1).

**Table 1:** Odds ratio and 95% confidence intervals of the top ranked model of Tasmanian devil (n = 18) and spotted-tailed quoll (n = 10) habitat selection.

Covariates	Tasmanian devils			Spotted-tailed quolls		
	Odds ratio	95% CI		Odds ratio	95% CI	
		Lower	Upper		Lower	Upper
Veg_Forest_	1.21	0.92	1.59	1.16	0.53	2.54
Veg_Grass_	0.84	0.65	1.09	1.53	0.68	3.44
Veg_Scrub/heath_	1.12	0.87	1.43	0.89	0.40	1.95
Road_Sealed_	8.51	7.10	10.17	1.60	1.14	2.23
Road_4WD_	2.93	2.41	3.56	0.96	0.71	1.27
Road_Unsealed_	6.22	4.80	8.04	2.18	1.29	3.65
Ecotone_Fence_	10.06	7.92	12.69	1.92	0.99	3.69
Ecotone_Pasture/cover_	2.64	2.21	3.13	1.84	1.07	3.12
Veg same_Yes_	1.00	0.88	1.13	1.15	0.93	1.41
*D*_*core*_	0.99	0.99	0.99	0.99	0.99	0.99

The reference level for vegetation type was pasture, the reference level for road and ecotone type were steps away from these features and the reference level for ‘Veg same’ was no.

The most parsimonious model for habitat selection of quolls was the full model (ω_i_ = 100%). The second best alternative model performed poorly in comparison with a ΔAIC of 14.48 for the model with all covariates except for Veg same. Quolls, regardless of sex, did not select natural vegetation over pasture (Table 1 and Supplementary Table S1). Quolls exhibited positive selection for sealed and unsealed roads compared to being away from roads (Table 1). They were almost twice as likely to be along a fence or along a pasture/cover ecotone than away from an ecotone (Table 1). Females showed a positive selection for all road and ecotone types, whereas males only showed a positive selection for unsealed roads (Supplementary Table S1). Results for females should be interpreted with caution due to the low sample size.

### Effect of habitat on movement rate and turn angles

**Figure 1:**
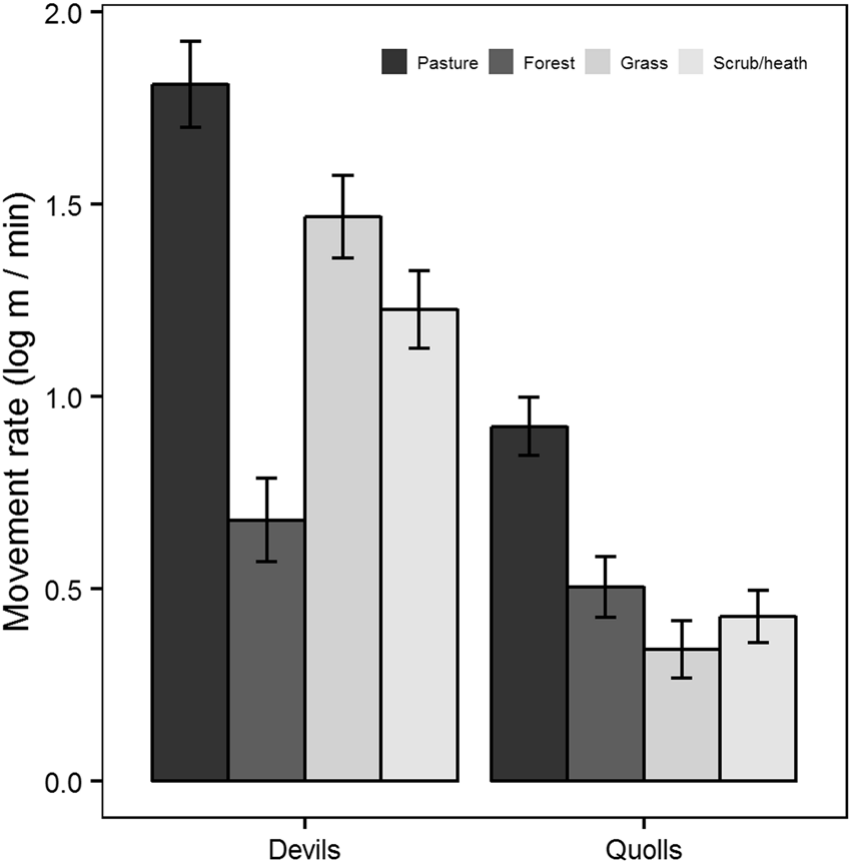
Mean (±standard error) movement rate based on 15 min fixes by vegetation type for devils (n = 18 devils, n = 9006 steps) and quolls (n = 10 quolls, n = 4462 steps).

**Figure 2:**
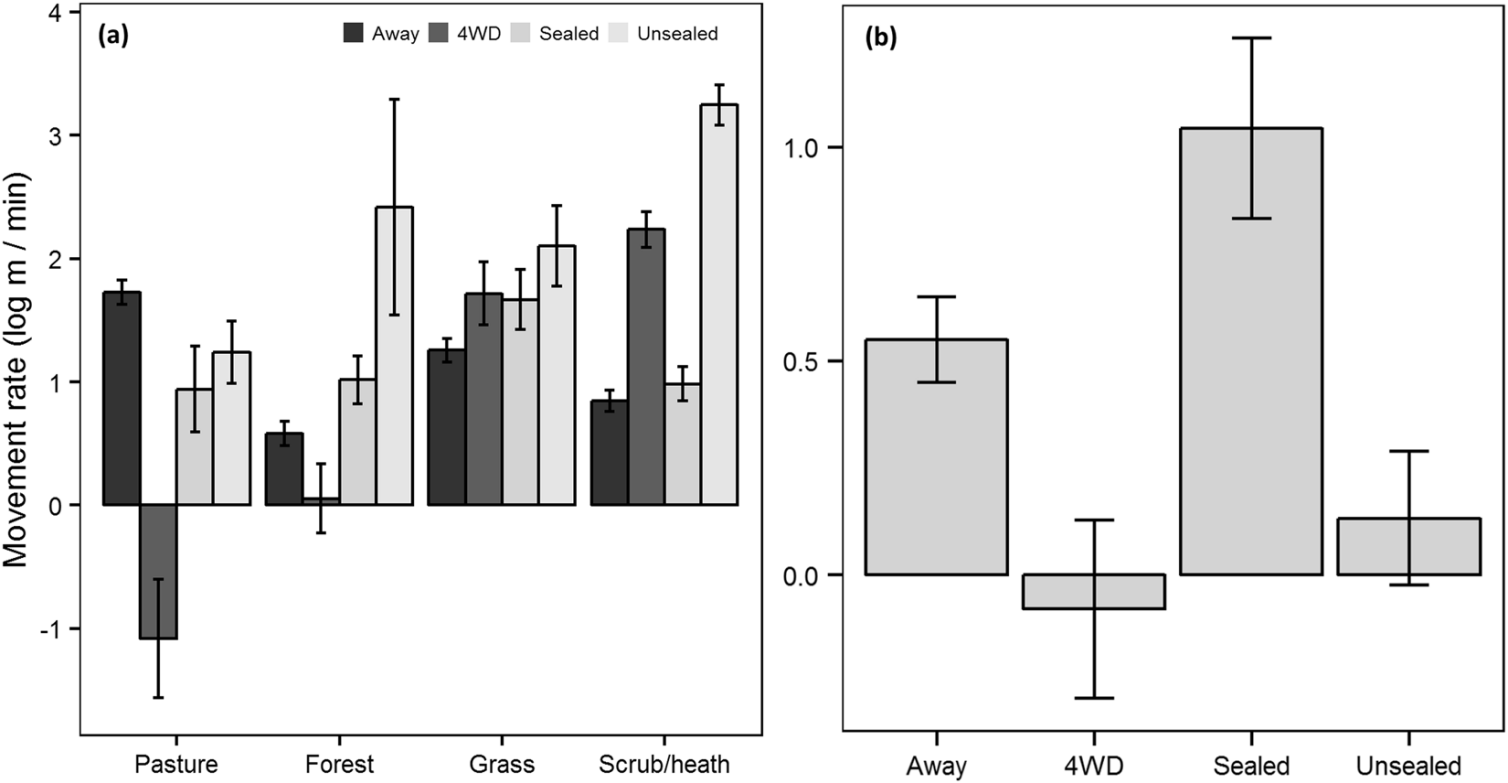
Mean (±standard error) movement rate based on 15 min fixes for (**a**) devils (n = 18 devils, n = 7127 steps) and (**b**) quolls (n = 10 quolls, n = 3965 steps) along roads compared to movement away them.

Both species moved more slowly in forest, grass (quolls only) and scrub/heath than in pasture (n = 9006 steps for devils and n = 4462 steps for quolls) (Fig. 1 and Table 2). The full model was the top model for devils along roads and the model with just road type was the top model for quolls (n = 7127 steps for devils and n = 3965 steps for quolls). Road type had a relative importance of 1 for both species (Table 2). Vegetation type and the interaction term had a relative importance of 1 for devils (Table 2). When the adjacent vegetation type was forest, grass or scrub/heath, devils moved more quickly along roads than in these vegetation types away from roads (except when forest was along 4WD tracks) (Fig. 2a). Devils moved more slowly through pasture adjacent to roads/tracks than through pasture away from roads/tracks (Fig. 2a). Quolls moved more slowly along 4WD tracks and unsealed roads but faster along the sealed road compared to movement away from roads (Fig. 2b).

**Table 2:** Model averaged results of GLMM analyses for movement rate (log m/min) for Tasmanian devils and spotted-tailed quolls.

	Fixed effects	Devils	RI	Quolls	RI
		Estimate ± SE		Estimate ± SE	
Vegetation type	Intercept	1.794 ± 0.112		1.041 ± 0.101	
	Veg		1		1
	Forest	−0.885 ± 0.078		−0.342 ± 0.082	
	Scrub/heath	−0.269 ± 0.065		−0.461 ± 0.073	
	Grass	−0.154 ± 0.080		−0.585 ± 0.072	
Road type	Intercept	1.724 ± 0.100		0.550 ± 0.099	
	Road		1		1
	4WD	−2.806 ± 0.477		−0.630 ± 0.187	
	Sealed	−0.783 ± 0.345		0.493 ± 0.188	
	Unsealed	−0.485 ± 0.248		−0.418 ± 0.127	
	Veg		1		na
	Forest	−1.144 ± 0.089			
	Scrub/heath	−0.879 ± 0.074			
	Grass	−0.468 ± 0.089			
	Road * Veg		1		na
	4WD*Forest	2.280 ± 0.551			
	Sealed*Forest	1.218 ± 0.393			
	Unsealed*Forest	2.320 ± 0.909			
	4WD*Grass	3.265 ± 0.539			
	Sealed*Grass	1.194 ± 0.420			
	Unsealed*Grass	1.333 ± 0.406			
	4WD*Scrub/heath	4.198 ± 0.495			
	Sealed*Scrub/heath	0.919 ± 0.366			
	Unsealed*Scrub/heath	2.886 ± 0.289			
Ecotone type	Intercept	1.504 ± 0.140		1.110 ± 0.129	
	Ecotone		1		1
	Pasture/cover	−0.494 ± 0.207		−1.206 ± 0.176	
	Fence	−0.289 ± 0.114		0.312 ± 0.498	
	Veg		1		1
	Cover	0.238 ± 0.077		−0.540 ± 0.076	
	Ecotone * Veg		0.58		1
	Pasture/cover*cover	−0.492 ± 0.268		0.635 ± 0.669	
	Fence*cover	0.283 ± 0.290		−0.653 ± 0.677	
	Random effect (ID)	Variance		Variance	
Vegetation type		0.41		0.26	
Road type		0.32		0.30	
Ecotone type		0.36		0.25	

SE = standard error and RI = relative importance of variables.

**Figure 3:**
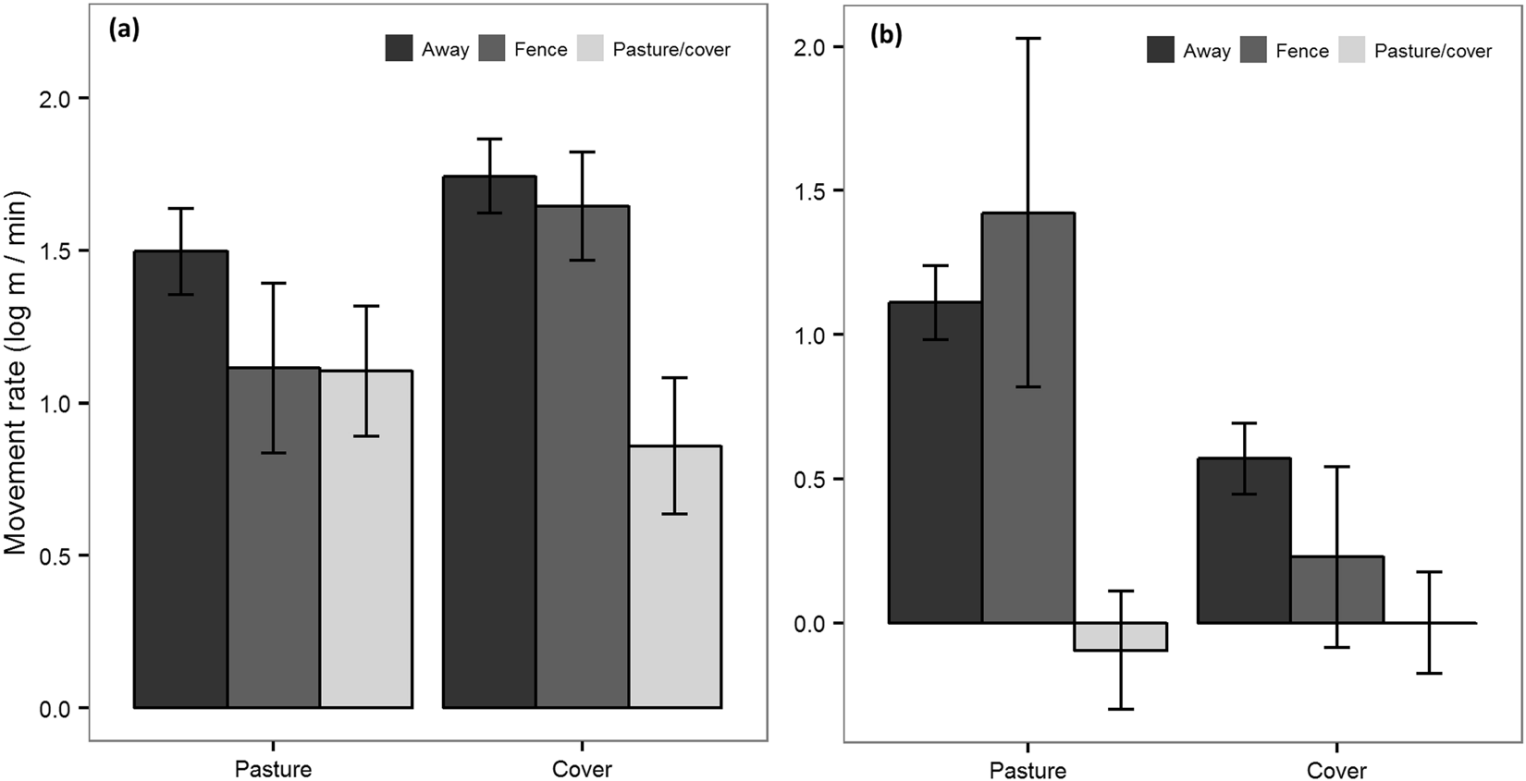
Mean (±standard error) movement rate based on 15 min fixes for (**a**) devils (n = 14 devils, n = 8448 steps) and (**b**) quolls (n = 5 quolls, n = 2816 steps) along ecotones compared to movement away them.

The final model set for movement rate in ecotones included two models for devils (n = 8448 steps); the full model (ω_i_ = 57%) and the model without the interaction term (ΔAIC = 0.62; ω_i_ = 42%). The most parsimonious model for quolls (n = 2816 steps) was the full model (ω_i_ = 93%). Ecotone type and vegetation type had a relative importance of 1 for both species (Table 2). The interaction between ecotone and vegetation had a relative importance of 1 for quolls and 0.58 for devils (Table 2). Both species moved slower along a pasture/cover ecotone compared to when they were moving through the landscape away from ecotones (Fig. 3). There was no difference in movement rate of devils and quolls along fences compared to movement away from any ecotone (Fig. 3). Quolls moved slower in cover compared to pasture when moving away from any ecotone and slower along the cover side of a fence (Fig. 3b and Table 2).

There was a wide distribution of turning angles for both species as demonstrated in the mean vector length (r) that ranged from 0.02–0.47 and the standard error of mean(s) that ranged from 1.82–141.95° (Table 3). Devils exhibited significant directional movement in all vegetation types (except in forest where movement was tortuous), and roads and ecotones and away from roads and ecotones (Table 3). They exhibited tortuous movement when they were moving in the cover side of an ecotone (Table 3). In contrast, quolls exhibited directional movement along sealed roads and when they were moving away from an ecotone and road (Table 3). Their movement was tortuous along fences, and in a pasture/cover ecotone, regardless of whether they were moving in the cover or pasture side (Table 3). They also exhibited tortuous movement along unsealed and 4WD tracks (Table 3). Quolls exhibited directional movement in pasture, grass and scrub/heath but tortuous movement in forest (Table 3).

**Table 3:** Summary statistics of Kuiper’s test for turn angle distribution near each vegetation, road and ecotone type for Tasmanian devils and spotted−tailed quolls.

		n	Mean turn angle (µ)	Mean vector length (r)	Standard error (s)	k	*P*
Devils	Vegetation						
	Pasture	1344	12.32°	0.27	4.08°	7.32	<**0.05**
	Forest	1359	289.74°	0.06	18.07°	3.07	>0.15
	Grass	1638	0.46°	0.25	3.97°	7.35	<**0.05**
	Scrub/heath	4665	355.18°	0.23	2.51°	13.15	<**0.05**
	Road						
	Away	5920	354.08°	0.12	4.25°	8.92	<**0.05**
	Sealed	518	354.10°	0.24	7.29°	5.88	<**0.05**
	Unsealed	297	1.27°	0.47	4.70°	6.21	<**0.05**
	4WD	392	356.87°	0.35	5.75°	5.30	<**0.05**
	Ecotone						
	Away	7924	0.89°	0.27	1.69°	17.88	<**0.05**
	Fence	287	9.38°	0.19	12.64°	2.99	<**0.05**
	Pasture/cover	237	340.34°	0.14	18.38°	2.02	<**0.05**
	Cover	111	325.98°	0.09	39.92°	1.40	>0.15
	Pasture	126	346.76°	0.19	19.14°	2.00	<**0.05**
Quolls	Vegetation						
	Pasture	1300	0.84°	0.27	4.12°	6.92	<**0.05**
	Forest	713	10.82°	0.02	66.63°	1.81	>0.15
	Grass	950	189.87°	0.08	15.84°	3.47	<**0.05**
	Scrub/heath	1499	210.38°	0.02	64.50°	2.92	<**0.05**
	Road						
	Away	5122	354.69°	0.04	13.08°	5.38	<**0.05**
	Sealed	97	1.12°	0.34	11.64°	2.75	<**0.05**
	Unsealed	232	352.35°	0.10	25.73°	2.37	>0.15
	4WD	96	112.13°	0.10	41.13°	1.32	>0.15
	Ecotone						
	Away	2590	356.93°	0.07	11.73°	4.29	<**0.05**
	Fence	31	352.96°	0.07	107.24°	0.79	>0.15
	Pasture/cover	195	8.24°	0.05	62.44°	1.30	>0.15
	Cover	118	19.63°	0.13	29.17°	1.52	>0.15
	Pasture	77	215.90°	0.08	55.77°	1.08	>0.15

Bold numbers indicates significant results (*P* < 0.05).

## Discussion

Our results provide a clear example of medium-sized carnivores favouring certain landscape features created by humans. Devils and quolls can be regarded as generalist carnivores exhibiting habitat plasticity and the ability to use edge habitats and linear features, traits that facilitate adaptation to fragmented landscapes^11, 13^. Despite differing ecomorphological specialisations, we demonstrate that both a pounce-pursuit predator that is a specialised scavenger (the devil) and an ambush predator that has morphological specialisations for arboreal activity (spotted-tailed quoll) can respond to anthropogenic modification of intact landscapes in ways that enable movement and foraging opportunities. They appear use the pasture/cover interface for foraging, roads for movement and foraging and fence lines (devils only) for movement.

The creation of additional linear features in landscapes may enhance what are already natural behaviours. Natural linear features occur in the form of animal trails, creek lines, and edges between closed and open vegetation types. Carnivores, including devils and quolls, use these to hunt, to move through vegetation (for example, following trails created by wombats (*Vombatus ursinus*) and macropods (M Jones, *pers. obs*.), and to position latrines in areas of high animal traffic^23, 24^. Human-altered landscapes and the linear features they contain are likely to benefit many species of medium-sized carnivores, including devils and quolls, by improving prey acquisition, either by enhancing opportunities for foraging or for travel to foraging areas^6, 25^.

Both species exhibited slow and tortuous movements in native forest vegetation, indicating that they are possibly foraging in these areas. Faster, straight movements across pasture suggest that while they use anthropogenic vegetation they do so for directional travel. A study, approximately 60 km north of our study site, found that female spotted-tailed quolls also exhibited linear and directional movement at night in pasture^21^, further supporting that quolls likely travel rather than forage in pasture. This study is limited to nocturnal movement patterns when both species are active, although quolls and occasionally devils also show diurnal activity^21^. The two species are likely to have different habitat requirements during the day. Devils den in intact habitats during the day, such as in the coastal scrub and patches of forest (G Andersen, unpublished data) and quolls also need complex vegetation structures for dens^21^.

The capacity of carnivores to respond positively to fragmentation is influenced not only by landscape structure but indirectly by the responses of prey species to fragmentation and the increased length of edges or ecotones between native vegetation and pasture^26^. On several continents, including Australia, vertebrate prey reach high population densities in fragmented landscapes that provide a diversity of habitat types^10, 27^. Many species, particularly the medium-sized herbivores that are often the major prey for medium-sized carnivores, favour edges where they can take refuge in intact native vegetation during the day and emerge through the cover/pasture ecotone to forage on pasture at night^28^. Edges thus provide carnivores with a rich prey source^10^ and prey individuals are especially vulnerable to predation when they cross the ecotone twice daily^29^. This high concentration of prey could support higher densities of medium-sized carnivores in fragmented agricultural landscapes^10, 30^. Slow and tortuous movement near the pasture/cover interface suggest that devils and quolls could be foraging. This interface provides ideal conditions to ambush their primary prey species, Tasmanian pademelon (*Thylogale billardierii*) and Bennett’s wallaby (*Macropus rufogriseus*) (G Andersen, unpublished data), as they emerge from their daytime refuges in cover through the pasture/cover ecotone to feed at night on pasture^28^. Troy (2014) also suggested that female quolls might be using this interface for foraging.

Roads are highly attractive to carnivores because they offer food, faster travel and sites for olfactory communication, although vehicle strike can also cause significant carnivore mortality^6, 7, 13^. Roads provide carcasses of animals killed by vehicles, as well as concentrations of herbivores attracted to feed on green grass that grows on roadside verges where run-off from the road provides additional moisture^31^. The linear path of the road and the edges between the road and the surrounding vegetation creates possibilities for ambushing prey. Reflecting their specialist scavenger niche, devils show a stronger selection than quolls for sealed roads, on which there is significant road mortality of prey species^32^. The fast and directed movement of both devils and quolls along the sealed road, where there are often road-killed animals suggests that they use spatial memory to revisit a profitable area. Quolls, which are ambush predators, show strong selection for unsealed roads, and their slow and tortuous movement patterns on unsealed roads and 4WD tracks suggest that they may be hunting. Unsealed roads, including 4WD tracks, create a linear open space which can act as a barrier to the movement of small mammals^33^ which may be vulnerable to carnivores if they linger in the adjacent vegetation.

Roads provide a linear corridor that could increase the distance travelled and extent of foraging by carnivores in a night. Commuting along roads to foraging areas is documented in several medium-sized carnivores, including red foxes, raccoons and striped skunks (*Mephitis mephitis*)^6^ and may explain road use by devils and quolls. Roads also offer prominent open locations for chemical communication and many carnivores, including wolves (*Canis lupus*)^34^ and black-backed jackals (*Canis mesomelas*)^35^, as well devils and quolls, deposit faeces and scent mark with para-anal or para-cloacal gland secretions on roads. Devils deposit faeces on all road types at the study site (G Andersen, *pers. obs*.), and quolls have been found to deposit faeces more frequently on maintained roads than on overgrown logging tracks or within the adjacent forest^36^.

Fences can impede movement of wildlife and contribute to habitat fragmentation^3, 37^. Devils travel extensively along fence lines and their low turning angles indicate directional travel. As the fences at our study site are designed to prevent macropods from moving onto pasture to graze at night, they have been built around two of the patches of forest; macropods rest and devils den in both of these patches during the day. Constructed of 150 mm × 80 mm wire mesh with an electric wire at ground level, they are impenetrable to macropods and devils alike, unless breached by animals such as wombats digging under the fence. It is, therefore, plausible that devils are traveling along fence lines looking for a way through (G Andersen, unpublished data). They could also use fence lines as roads to quickly move through the landscape. In addition, prey species become entangled and die in these fences and devils have been observed scavenging along them (G Andersen, unpublished data).

While we have demonstrated the positive response of devils and quolls to moderate landscape fragmentation, both fences and roads also have negative effects on carnivores and other wildlife. Understanding how fences affect movement of carnivores and other wildlife, and working with landowners to develop structures to facilitate movement of key species through fences, is important to ensure connectivity among populations. The impact of roads on wildlife is of global concern^4^ and many carnivores are highly susceptible to mortality from vehicles^5^. Devils and quolls are both listed on the IUCN Red List, devils as Endangered^19^ and spotted-tailed quolls Near Threatened^20^. Road mortality is a demonstrated cause of local population decline for devils and eastern quolls^38^. Spotted-tailed quolls are also subject to road mortality^32^. Potential measures to reduce road mortality include wildlife crossing structures^39^, and virtual fences, consisting of flashing light and sound alarm units set out at 100 m intervals which are triggered by the headlights of approaching vehicles, which show promise in reducing road mortality of wildlife^40^.

Medium-sized carnivores, more than large carnivores, are thought to be better able to adapt to anthropogenic landscape alteration and fragmentation, probably because of their smaller size and area requirements and generalist ecologies^12^. Our study demonstrates that two species of medium-sized carnivores with contrasting ecomorphological specialisations and hunting modes can adapt to moderate landscape modification in their native range in Tasmania, Australia. Such adaptability is well known in successful invaders such as red foxes but also in some medium-sized carnivores in their native range. Our results contribute to an increasing body of literature that demonstrates such adaptability is widespread across different taxa (including marsupial as well as placental carnivores), continents and ecomorphological types (specifically hypercarnivores and scavengers in this study, as well as generalist insectivore/carnivores). Agents of mortality for carnivores abound in anthropogenic landscapes, however, and include collisions with vehicles, persecution by humans^1, 5^ and restrictions on movement from fences^37^. Carnivores also need species-specific minimum areas of structurally complex vegetation for den sites and for refuge^41^. Retaining linear remnants and small patches of native vegetation in agricultural landscapes is important to facilitate animal movement through the matrix^42^. Identifying thresholds in the degree of anthropogenic landscape modification that carnivore species can benefit from and persist within, with respect to denning and foraging habitat, and ensuring that these are not exceeded, will aid the ongoing conservation of carnivores in these habitats.

## Material and Methods

### Study area

**Figure 4:**
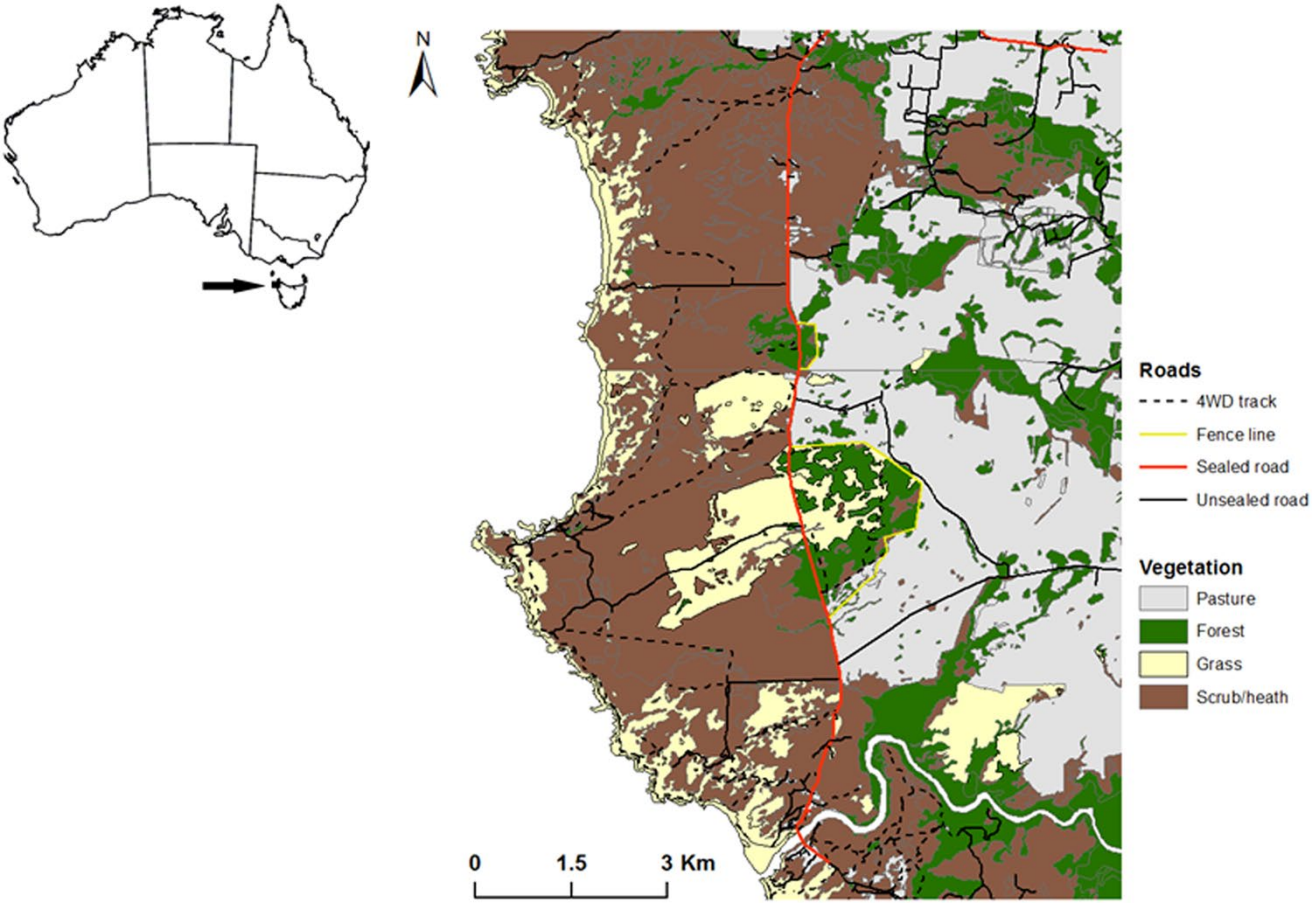
The study area in northwest Tasmania, Australia. Vegetation and road types are displayed. Esri ArcGIS 10.2 (https://esriaustralia.com.au/products-arcgis-software-102) and Microsoft Powerpoint (Microsoft, Seattle, WA) was used to create this figure. The vegetation layer was created using TASVEG 3.0 (Department of Primary Industries, Parks, Water and Environment, Tasmania, TASVEG 3.0, Sourced on 13^th^ October 2013, http://dpipwe.tas.gov.au/conservation/flora-of-tasmania/monitoring-and-mapping-tasmanias-vegetation-(tasveg)/tasveg-the-digital-vegetation-map-of-tasmania) and the road layer using LIST transport (Department of Primary Industries, Parks, Water and Environment, Tasmania, LIST transport, Sourced 27^th^ of March 2015, https://data.gov.au/dataset/list-transport-segments).

The study area covered approximately 100 km^2^ of the Arthur-Pieman Conservation Area (Fig. 4). It encompassed native and modified vegetation, and a network of roads consisting of a 12 km section of sealed road running through the centre of the area and gravel, dirt and 4WD minor roads and tracks. Areas to the west of the sealed road were dominated by coastal scrub/heath (*Leptospermum scoparium*, *Acacia longifolia*, *Melaleuca squarrosa and Leucopogon collinus*) and moorland (*Gymnoschoenus sphaerocephalus*). The east was a mosaic of forest (*Eucalyptus obliqua*, *Eucalyptus nitida*, *Melaleuca ericifolia* and *Leptospermum lanigerum*) and agricultural land with cattle grazing on pasture. The climate is temperate, with monthly mean temperatures ranging from 9.4–16.1 °C, and mean annual rainfall of 1069 mm^43^.

### GPS telemetry

Animals were trapped in PVC pipe traps (diameter 315mm × length 875mm) and fitted with GPS collars (Quantum 4000E, Telemetry Solutions Ltd, Concord, USA) from November 2012-February 2013 and October 2013-January 2014. These periods covered times when both species are in late lactation with young in dens and when they are weaned (devils early February; quolls December). Collars weighed 185 g (devils) and 60 g (quolls), less than 3% of body weight. Animals were not sedated before collaring. Collars were fastened with corrodible links which degrade over time and eventually allow the collar to fall off^44^. Collar schedules were set to collect simultaneous fixes of both species. Because devils are mainly active at night^22^ collars were programmed to record a GPS fix every 15 min from 2030 to 0630 hours to conserve battery and to extend the duration of data collection. This study was approved by the Animal Ethics Committee at the University of Tasmania, Australia. The methods were carried out in accordance with the approved guidelines and regulations.

### GPS data screening

Animal locations obtained through GPS contain errors due to missing location fixes or location errors of successfully acquired fixes^45^. These must be removed. First, we visually screened for GPS errors and spikes using ‘adehabitatLT’^46^ in R version 3.1.3 (R Development Core Team 2015). Second, because Horizontal Dissolution of Precision (HDOP) is related to location error^47^, we determined an appropriate HDOP threshold to ensure that positional accuracy was similar to the resolution of our vegetation data (±5 m). A low HDOP value represents a higher level of precision as the satellites used to generate the fix are widely dispersed across the sky^45^. We placed a stationary test collar in our study site that was left recording 15 minute fixes for two days. The Euclidean distance between each recorded location and the true location, which was measured with a handheld GPS unit (Garmin GPSMAP 78, Garmin Ltd, USA) was calculated and we noted the corresponding HDOP value. The mean linear error for each HDOP value (prior to fix clean up) was then calculated and we retained only GPS fixes with a dilution of precision (HDOP) of <7. Mean GPS error was 7.12 ± 0.41 m.

To investigate whether vegetation type influenced the success rate of location fixes obtained by the GPS units in tracking collars, we conducted two tests. First, we placed a stationary test collar, which was programmed to record 15 minute fixes for two nights, in each of the four vegetation types. Second, we plotted the temperature (ambient temperature measured by GPS collars) of zero fixes (GPS failed to record a fix) and active fixes (step length >20 m for 15 min fixes) for both species. Devils have a minimum body temperature of 31.3 °C^22^, therefore if the temperature of zero fixes is >31.3 °C we have assumed that the devil is resting on the collar (most likely in a den) and the lack of a fix was not due to vegetation type. The minimum body temperature of spotted-tailed quolls is unknown but we assumed it to be the same as eastern quolls (*Dasyurus viverrinus*), which is 33.5 °C^22^. A study by Jones *et al*. (1997)^22^ and Andersen *et al*. (unpublished data) found that devils spend half of the night resting in a den, where the GPS collar will be unable to get a fix. Therefore, we expect a high number of zero fixes associated with high temperatures recorded on the collars for devils. We conducted a test of the accuracy of the GPS units in recording temperature at higher and lower ambient temperatures, and during zero fixes when the collar could not obtain a satellite connection. Ten GPS collars were programmed to record fixes every 10 minutes for an hour at various temperatures and ambient temperature was measured with a mercury thermometer.

### Habitat selection

A vegetation and road map of the study site was created in ArcGIS 10.2 (ESRI, Redlands, CA) using the Tasmanian vegetation mapping spatial database TASVEG 3.0^48^ and LIST Transport^49^, and verified through high resolution (1:2000) digital orthophotographs (Department of Primary Industries, Parks, Water and Environment, Tasmania, Australia). We combined structurally similar vegetation communities into four categories and categorized roads and tracks according to structure and amount of traffic (Table 4). Home-range (the area containing 100% of the locations) and core-area size (the area containing 50% of the locations) for each animal were determined using movement-based kernel density estimation methods (MKDE)^50^. As MKDE benefits from serial autocorrelated data^50^ we used data at 15 min intervals.

**Table 4:** Description of the habitat covariates used in analyses of anthropogenic habitat use of Tasmanian devils and spotted-tailed quolls.

hbp	Variable	Description
Vegetation type	Pasture	Grass paddocks with cattle (height <0.5 m)
	Forest	*Eucalyptus obliqua*, *E. nitida*, *Melaleuca ericifolia* and *M. squarrosa* swamp forest
	Scrub/heath	*Leptospermum lanigerum*, costal heathland, *Acacia longifolia* and *M. squarrosa* scrub
	Grass	Native grassland, buttongrass moorland and wetlands (height > 0.5 m)
Road type	Away	Steps not on a road
	Sealed	The main road that runs through the centre of the study site
	Unsealed	Receives moderate amount of traffic
	4WD	Receives limited or no traffic
Ecotone type	Away	Steps not in an ecotone
	Pasture/cover	Interface between vegetation and pasture
	Fence	Wildlife-proof fences that were placed on ecotones between cover and pasture

We used step selection functions (SSF)^51^ based on sequential successful 15 minute fixes to examine habitat selection for devils and quolls and for each sex. For each observed step, 14 random steps were generated within each animal’s 100% MKDE home range polygon, to compare the habitat or feature selected by the animal to a range of ‘available’ habitats/features. To create the random steps, we first created probability distributions of a devil or quolls step length and turn angle based on 15 minute fixes and then used these probability distributions to randomly select 14 points. Random points were then assigned the habitat variable of interest using the vegetation map. Home range polygons did not include the ocean, so no random fixes were located at implausible locations. We created a 20 m buffer either side of a road or track and categorised steps that fell within this buffer to be in the vicinity of and therefore potentially influenced by the road. Similarly, we created a 20 m buffer either side of an interface between vegetation and pasture (‘pasture/cover’) and either side of wildlife-proof fences (‘fence’) to account for any influence of ecotones. We used steps ‘away’ from an ecotone or road type as a reference level (Table 4), as we aimed to characterize changes in habitat selection or movement rate near these features to habitat selection/movement rate away from them.

Models were created using case-control logistic regression^52^ using the package ‘survival’^53^ in the statistical programme R version 3.1.3^54^. The animal’s selection is measured as an odds ratio, which is a measure of effect size and describes the strength of association between two binary data values. It represents the magnitude of change in the odds selection for each unit of the predictor variable. Devil and quoll individual ID was treated as a random effect in models. We included vegetation type, road type, ecotone type and distance to the nearest core area polygon edge (‘*D*_core_’) (continuous variable) as parameters in the model selection analysis. Because the animal’s selection of vegetation type in successive GPS fixes may be dependent on the last vegetation type it was in, we fitted a binary ‘carry-over’ variable (‘Veg same’), describing whether the vegetation type was equal to the previous fix. We tested for collinearity among explanatory variables using chi-squared tests. An information theoretic framework was used to rank competing models based on Akaike’s information criterion corrected for small sample sizes (AICc)^55^; models within a ΔAICc of two were considered the most plausible, with substantial empirical support. In addition, we used Akaike weights (ω_i_) to gauge the relative importance of variables that influence habitat selection.

### Effect of habitat on movement rate and turn angles

We constructed separate generalized linear mixed models (GLMMs) for each of the three habitat covariates described in Table 1 for devils and quolls based on 15 minute fixes. Movement between two successive locations was defined as a step and average step rate per minute was calculated. For all analyses, we log-transformed (log_e_) movement rate (m/min) to meet the assumptions of normality and fitted it as the response term. Following Troy (2014), active fixes (step length ≥ 21 m) and inactive fixes (step length ≤ 20 m) were included in analyses but long periods of rest (≥6 hours where all step lengths and the total net displacement ≤ 20 m) were excluded from analysis^21^. Inclusion of inactive fixes allows for short periods of rest and prey handling. Devil and quoll individual ID was included as a random variable to account for repeated observations of the same individual. In the devil and quoll model containing ecotone type, we combined forest, grass and scrub/heath into a category called ‘cover’ to contrast movement in the vegetation side of an ecotone to movement in the pasture side of an ecotone. Vegetation type (‘cover’ or ‘pasture’), ecotone type and their interaction were included as fixed factors. The devil road type model included vegetation type, road type and their interaction as fixed factors. The quoll road type model included only road type, as there were not enough steps in some vegetation types near roads to include vegetation type. Only steps that had both locations within an ecotone, road type or vegetation type were used in all models. Statistical analyses for movement rates were undertaken using the ‘nlme’ package^56^ in R version 3.1.3^54^. Parameter estimates were averaged across the final model set and the relative importance of predictor variables was assessed by summing Akaike weights across all models in which the variable appeared^55^.

We examined the turning angle of steps within a vegetation type, road type and ecotone type using circular statistics^57^ using the ‘circular’ package^58^ in R version 3.1.3^54^. We also examined the turning angle of steps within the cover and pasture side of the pasture/cover ecotone. Turn angles were calculated as the clockwise angle relative to the movement trajectory. We computed the mean turning angle (µ), mean vector length (r) and standard error (s) for the distribution of turning angles. The mean vector (r) is a measure of directionality for circular data that ranges from 0 (angles are distributed randomly) to 1 (all angles are identical). We tested for directionality of movement within each ecotone type, road type and vegetation type using Kuiper’s test of uniformity. The null hypothesis in the Kuiper’s test states that angles are uniformly distributed around the circle, which corresponds to tortuous movement. Directional travel is commonly associated with movement behaviours such as movement between patches, maintenance of territory, and mate seeking behaviours, whereas tortuous movement is often indicative of foraging^59, 60^.

### Data availability

The datasets generated during and analysed during the current study are available from the corresponding author on reasonable request.

## Electronic supplementary material

Supplementary material

## References

[1] Woodroffe R (2000). Predators and people: using human densities to interpret declines of large carnivores. Anim. Conserv..

[2] Purvis A, Gittleman JL, Cowlishaw G, Mace GM (2000). Predicting extinction risk in declining species. Proc. R. Soc. B..

[3] Cozzi G, Broekhuis F, McNutt JW, Schmid B (2013). Comparison of the effects of artificial and natural barriers on large African carnivores: implications for interspecific relationships and connectivity. J. Anim. Ecol..

[4] Fahrig L, Rytwinski T (2009). Effects of roads on animal abundance: an empirical review and synthesis. Ecol. Soc..

[5] Cervinka J, Riegert J, Grill S, Salek M (2015). Large-scale evaluation of carnivore road mortality: the effect of landscape and local scale characteristics. Mammal Research.

[6] Frey SN, Conover MR (2006). Habitat use by meso-predators in a corridor environment. J. Wildl. Manag..

[7] Dickie M, Serrouya R, McNay RS, Boutin S (2016). Faster and farther: wolf movement on linear features and implications for hunting behaviour. J. Appl. Ecol..

[8] Lambertucci SA, Speziale KL, Rogers TE, Morales JM (2009). How do roads affect the habitat use of an assemblage of scavenging raptors?. Biodivers. Conserv..

[9] Knight RL, Kawashima JY (1993). Responses of raven and red-tailed hawk populations to linear right-of-ways. J. Wildl. Manag..

[10] Salek M, Kreisinger J, Sedlacek F, Albrecht T (2010). Do prey densities determine preferences of mammalian predators for habitat edges in an agricultural landscape?. Landsc. Urban Plann..

[11] Cervinka J, Salek M, Pavluvcik P, Kreisinger J (2011). The fine-scale utilization of forest edges by mammalian mesopredators related to patch size and conservation issues in Central European farmland. Biodivers. Conserv..

[12] Crooks KR (2002). Relative sensitivities of mammalian carnivores to habitat fragmentation. Conserv. Biol..

[13] Bateman PW, Fleming PA (2012). Big city life: carnivores in urban environments. J. Zool..

[14] Hoffmann M (2010). The impact of conservation on the status of the world’s vertebrates. Science.

[15] Johnson DH (1980). The comparison of usage and availability measurements for evaluating resource preference. Ecology.

[16] Jones ME, Stoddart DM (1998). Reconstruction of the predatory behaviour of the extinct marsupial thylacine (*Thylacinus cynocephalus*). J. Zool..

[17] Jones ME, Barmuta LA (1998). Diet overlap and relative abundance of sympatric dasyurid carnivores: a hypothesis of competition. J. Anim. Ecol..

[18] Hawkins CE (2006). Emerging disease and population decline of an island endemic, the Tasmanian devil *Sarcophilus harrisii*. Biol. Conserv..

[19] Hawkins, C. E., McCallum, H., Mooney, N., Jones, M. & Holdsworth, M. *Sarcophilus harrisii. The IUCN Red List of Threatened Species 2008*, (2008) (2/10/16).

[20] Burnett, S. & Dickman, C. *Dasyurus maculatus. The IUCN Red List of Threatened Species 2008*, (2008) (12/10/2016).

[21] Troy, S. N. *Spatial ecology of the Tasmanian spotted-tailed quoll*. Ph.D. thesis, University of Tasmania, Hobart (2014).

[22] Jones ME, Grigg GC, Beard LA (1997). Body temperatures and activity patterns of Tasmanian devils (*Sarcophilus harrisii*) and eastern quolls (*Dasyurus viverrinus*) through a subalpine winter. Physiol. Zool..

[23] Ruibal M, Peakall R, Claridge A (2010). Socio-seasonal changes in scent-marking habits in the carnivorous marsupial *Dasyurus maculatus* at communal latrines. Aust. J. Zool..

[24] Hopcraft JGC, Sinclair ARE, Packer C (2005). Planning for success: Serengeti lions seek prey accessibility rather than abundance. J. Anim. Ecol..

[25] Knopff AA, Knopff KH, Boyce MS, St Clair CC (2014). Flexible habitat selection by cougars in response to anthropogenic development. Biol. Conserv..

[26] Mortelliti A, Boitani L (2008). Interaction of food resources and landscape structure in determining the probability of patch use by carnivores in fragmented landscapes. Landsc. Ecol..

[27] Cervinka J, Salek M, Padysakova E, Smilauer P (2013). The effects of local and landscape-scale habitat characteristics and prey availability on corridor use by carnivores: a comparison of two contrasting farmlands. J. Nat. Conserv..

[28] Le Mar K, McArthur C (2005). Comparison of habitat selection by two sympatric macropods, *Thylogale billardierii* and *Macropus rufogriseus rufogriseus*, in a patchy eucalypt-forestry environment. Austral. Ecol..

[29] Nielsen, A. *The potential impact of Tasmanian devil (Sarcophilus harrisii) decline on prey behaviour*. Bachelor of Science (Honours) thesis, University of Tasmania (2009).

[30] Saunders, A. *The occupance of native and introduced Tasmanian carnivores in intact and fragmented landscapes*. BSc Honours thesis (2012).

[31] Klöcker U, Croft DB, Ramp D (2006). Frequency and causes of kangaroovehicle collisions on an Australian outback highway. Wildl. Res..

[32] Hobday AJ, Minstrell ML (2008). Distribution and abundance of roadkill on Tasmanian highways: human management options. Wildl. Res..

[33] Rico A, Kindlmann P, Sedlacek F (2007). Barrier effects of roads on movements of small mammals. Folia Zool..

[34] Barja I, de Miguel FJ, Barcena F (2004). The importance of crossroads in faecal marking behaviour of the wolves (*Canis lupus*). Naturwissenschaften.

[35] Hayward MW, Hayward GJ (2010). Potential amplification of territorial advertisement markings by black-backed jackals (Canis mesomelas). Behaviour.

[36] Burnett, S. E. *Ecology and conservation status of the northern spot-tailed quoll, Dasyurus maculatus with reference to the future of Australia’s marsupial carnivores*. Ph.D. thesis, James Cook University (2001).

[37] Gates, C. C. *et al*. The Influence of Land Use and Fences on Habitat Effectiveness, Movements and Distribution of Pronghorn in the Grasslands of North America In *Fencing for Conservation: Restriction of Evolutionary Potential or a Riposte to Threatening Processes?* (ed. Somers, J. M. & Hayward, M.), 277–294 (Springer New York, 2012).

[38] Jones ME (2000). Road upgrade, road mortality and remedial measures: impacts on a population of eastern quolls and Tasmanian devils. Wildl. Res..

[39] Grilo, C., Smith, D. J. & Klar, N. Carnivores: struggling for survival in roaded landscapes in *Handbook of road ecology* (ed. Van der Ree, R., Smith, D. J. & Grilo, C.), 300–312 (Wiley-Blackwell, 2015).

[40] Potts, J. M. Analysis of road kill data collected at Arthur River to test the efficacy of a virtual fence in reducing road kill. The Analytical Edge Statistical Consulting (2015).

[41] Sunquist, M. E. & Sunquist, F. Changing landscapes: consequences for carnivores in *Carnivore Conservation* Vol. 5 *Conservation Biology* Series(ed. Gittleman, J. L., Funk, S. M., Macdonald, D. W., & Wayne, R. K.), 399–418 (Cambridge University Press, 2001).

[42] Taylor PD, Fahrig L, Henein K, Merriam G (1993). Connectivity is a vital element of landscape structure. Oikos.

[43] Australian Bureau of Meteorology. *Australian Bureau of Meteorology*, (2016) (4/2/2016).

[44] Thalmann S (2013). Evaluation of a degradable time-release mechanism for telemetry collars. Aust. Mammal..

[45] Bjorneraas K, Van Moorter B, Rolandsen CM, Herfindal I (2010). Screening Global Positioning System location data for errors using animal movement characteristics. J. Wildl. Manag..

[46] Calenge C (2006). The package “adehabitat” for the R software: A tool for the analysis of space and habitat use by animals. Ecol. Model..

[47] D’Eon RG (2003). Effects of a stationary GPS fix-rate bias on habitat selection analyses. J. Wildl. Manag..

[48] Department of Primary Industries, Parks, Water and Environment. TASVEG 3.0 Released November 2013 *Tasmanian Vegetation Monitoring and Mapping Program, Resource Management and Conservation Division*, Hobart, Australia. http://dpipwe.tas.gov.au/conservation/flora-of-tasmania/monitoring-and-mapping-tasmanias-vegetation-(tasveg)/tasveg-the-digital-vegetation-map-of-tasmania (2013).

[49] Department of Primary Industries, Parks, Water and Environment. LIST transport *Land Tasmania*, Hobart, Australia. https://data.gov.au/dataset/list-transport-segments (2009).

[50] Benhamou S, Cornelis D (2010). Incorporating movement behavior and barriers to improve kernel home range space use estimates. J. Wildl. Manag..

[51] Fortin D (2005). Wolves influence elk movements: behavior shapes a trophic cascade in Yellowstone National Park. Ecology.

[52] Gail MH, Lubin JH, Rubinstein LV (1981). Likelihood Calculations for Matched Case-Control Studies and Survival Studies with Tied Death Times. Biometrika.

[53] Therneau, T. A package for survival analysis in R. version 2.38, http://CRAN.R-project.org/package=survival (2015).

[54] R Core Team. R: a language and environment for statistical computing *R Foundation for Statistical Computing*, Vienna, Austria. www.r-project.org (2015).

[55] Burnham, K. P. & Anderson, D. R. Model selection and multi-model inference: a practical information-theoretic approach. 2nd edn, (Springer, 2002).

[56] Pinheiro, J., Bates, D., DebRoy, S., Sarkar, D. & Team., R. C. nlme: linear and nonlinear mixed effects models. R package version 3.1–120, http://CRAN.R-project.org/package=nlme (2015).

[57] Batschelet, E. *Circular Statistics in Biology*. (Academic Press, 1981).

[58] Agostinelli, C. & Lund, U. R package ‘circular’: Circular Statistics (version 0.4-7), https://r-forge.r-project.org/projects/circular (2013).

[59] Nams VO, Bourgeois M (2004). Fractal analysis measures habitat use at different spatial scales: an example with American marten. Can. J. Zool..

[60] Fuller AK, Harrison DJ (2010). Movement paths reveal scale-dependent habitat decisions by Canada lynx. J. Mammal..

