# Supplementary material for: Use of anthropogenic linear features by two medium-sized carnivores in reserved and agricultural landscapes
Source: Sci Rep. 2017 Sep 14;7:11624. doi: 10.1038/s41598-017-11454-z (PMC5599595; doi:10.1038/s41598-017-11454-z)
Supplement: Supplementary file 1 — Supplementary material [file 41598_2017_11454_MOESM1_ESM.doc]

**Supplementary Information**

**Use of anthropogenic linear features by two medium-sized carnivores in reserved and agricultural landscapes**

Georgina E. Andersen^1*^, Christopher N. Johnson^1^, Leon A. Barmuta^1^ and Menna E. Jones^1^

^1^ School of Biological Sciences, University of Tasmania, Private Bag 55, Hobart, TAS 7001, Australia

* Corresponding author: Georgina Andersen: [georgina.andersen@utas.edu.au](mailto:georgina.andersen@utas.edu.au)


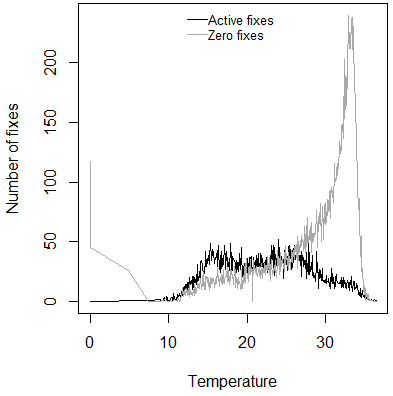

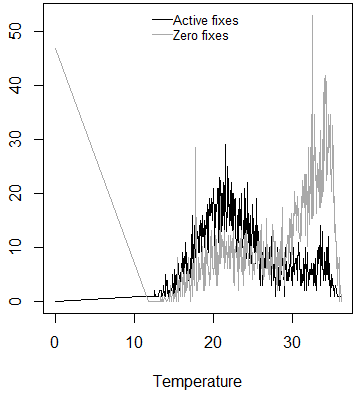


**Fig. S1.** Temperature of active (step length >20m for 15min fixes) and zero fixes for (a) devils and (b) quolls.

**Table S1.** Odds ratio and 95% confidence intervals of the top ranked model of habitat selection by sex. The reference vegetation type was pasture, the reference road and ecotone type were steps away from these features and the reference for ‘veg same’ was no.

|  | Tasmanian devils | | | | | | Spotted-tailed quolls | | | | | |
| --- | --- | --- | --- | --- | --- | --- | --- | --- | --- | --- | --- | --- |
|  | Males | | | Females | | | Males | | | Females | | |
|  | Odds ratio | 95% CI | | Odds ratio | 95% CI | | Odds ratio | 95% CI | | Odds ratio | 95% CI | |
| Covariates |  | Lower | Upper |  | Lower | Upper |  | Lower | Upper |  | Lower | Upper |
| Veg_Forest_ | 1.46 | 1.02 | 2.08 | 1.11 | 0.69 | 1.46 | 2.15 | 0.73 | 6.33 | 0.97 | 0.56 | 1.24 |
| Veg_Grass_ | 0.88 | 0.65 | 1.20 | 0.82 | 0.53 | 1.25 | 1.49 | 0.32 | 6.96 | 1.83 | 0.95 | 3.53 |
| Veg_Scrub/heath_ | 1.09 | 0.73 | 1.61 | 1.15 | 0.84 | 1.59 | 1.18 | 0.25 | 5.66 | 0.55 | 0.25 | 1.18 |
| Road_Sealed_ | 7.42 | 5.70 | 9.66 | 9.70 | 8.16 | 11.52 | 1.40 | 0.84 | 2.32 | 2.16 | 1.55 | 3.00 |
| Road_4WD_ | 2.98 | 2.17 | 4.07 | 2.89 | 2.27 | 3.69 | 0.91 | 0.62 | 1.33 | 1.26 | 1.02 | 1.54 |
| Road_Unsealed_ | 4.93 | 3.82 | 6.38 | 7.92 | 5.35 | 11.71 | 1.76 | 1.31 | 2.36 | 2.70 | 1.04 | 6.85 |
| Ecotone_Fence_ | 8.75 | 5.48 | 13.94 | 11.43 | 9.58 | 13.62 | 1.39 | 0.37 | 5.30 | 2.53 | 1.89 | 3.37 |
| Ecotone_Pasture/cover_ | 2.42 | 1.88 | 3.10 | 2.88 | 2.36 | 3.52 | 1.20 | 0.61 | 2.38 | 2.49 | 1.84 | 3.39 |
| Veg same_Yes_ | 1.03 | 0.82 | 1.28 | 0.98 | 0.87 | 1.10 | 1.16 | 0.85 | 1.57 | 1.10 | 0.74 | 1.62 |
| *D_core_* | 0.99 | 0.99 | 0.99 | 0.99 | 0.99 | 0.99 | 0.99 | 0.99 | 0.99 | 0.99 | 0.99 | 0.99 |
